# Immune-Response Patterns and Next Generation Sequencing Diagnostics for the Detection of Mycoses in Patients with Septic Shock—Results of a Combined Clinical and Experimental Investigation

**DOI:** 10.3390/ijms18081796

**Published:** 2017-08-18

**Authors:** Sebastian O. Decker, Annette Sigl, Christian Grumaz, Philip Stevens, Yevhen Vainshtein, Stefan Zimmermann, Markus A. Weigand, Stefan Hofer, Kai Sohn, Thorsten Brenner

**Affiliations:** 1 Department of Anesthesiology, Heidelberg University Hospital, 110, Im Neuenheimer Feld, D-69120 Heidelberg, Germany; Sebastian.Decker@med.uni-heidelberg.de (S.O.D.); Annette.Sigl@gmx.de (A.S.); Markus.Weigand@med.uni-heidelberg.de (M.A.W.); 2 Fraunhofer IGB, 12, Nobelstraße, D-70569 Stuttgart, Germany; Christian.Grumaz@igb.fraunhofer.de (C.G.); philip.stevens@noscendo.com (P.S.); yevhen.vainshtein@igb.fraunhofer.de (Y.V.); Kai.Sohn@igb.fraunhofer.de (K.S.); 3 Noscendo GmbH, 9, Meitnerstraße, D-70563 Stuttgart, Germany; 4 Department of Infectious Diseases, Medical Microbiology and Hygiene, Heidelberg University Hospital, 324, Im Neuenheimer Feld, D-69120 Heidelberg, Germany; Stefan.Zimmermann@med.uni-heidelberg.de; 5 Department of Anesthesiology, Westpfalzklinikum, 1, Hellmut-Hartert-Straß, D-67655 Kaiserslautern, Germany; shofer@westpfalz-klinikum.de

**Keywords:** mid-regional proadrenomedullin, interleukin-17A, β-d-glucan, next-generation sequencing, mycoses, sepsis, septic shock

## Abstract

Fungi are of increasing importance in sepsis. However, culture-based diagnostic procedures are associated with relevant weaknesses. Therefore, culture- and next-generation sequencing (NGS)-based fungal findings as well as corresponding plasma levels of β-d-glucan, interferon gamma (INF-γ), tumor necrosis factor alpha (TNF-α), interleukin (IL)-2, -4, -6, -10, -17A, and mid-regional proadrenomedullin (MR-proADM) were evaluated in 50 septic patients at six consecutive time points within 28 days after sepsis onset. Furthermore, immune-response patterns during infections with *Candida* spp. were studied in a reconstituted human epithelium model. In total, 22% (*n* = 11) of patients suffered from a fungal infection. An NGS-based diagnostic approach appeared to be suitable for the identification of fungal pathogens in patients suffering from fungemia as well as in patients with negative blood cultures. Moreover, MR-proADM and IL-17A in plasma proved suitable for the identification of patients with a fungal infection. Using RNA-seq., adrenomedullin (ADM) was shown to be a target gene which is upregulated early after an epithelial infection with *Candida* spp. In summary, an NGS-based diagnostic approach was able to close the diagnostic gap of routinely used culture-based diagnostic procedures, which can be further facilitated by plasmatic measurements of MR-proADM and IL-17A. In addition, ADM was identified as an early target gene in response to epithelial infections with *Candida* spp.

## 1. Introduction

Sepsis, defined as a life-threatening organ dysfunction caused by a dysregulated host response to infection [1], is most frequently a result of a bacterial infection [2,3,4], whereas those of a fungal or viral nature are less common [5,6,7]. Accordingly, fungemia is only present in 3% of unselected sepsis cases [5,8,9]. Nevertheless, fungi are one of the most isolated species recovered from abdominal foci in peritonitis [10,11] and numerous patients develop fungal colonization during their hospital stay [5]. Although the group of infected patients appears to be small, the number of severe fungal infections is escalating due to an increase in the number of immunocompromised patients, a more aggressive surgical therapy in older patients with relevant co-morbidities, and an increasing number of oncologic diseases [5,8,12,13]. Within this context, three fungal species seem to be of most relevance in Europe: *Candida albicans* (*C. albicans*), *Candida glabrata* (*C. glabrata*), and *Aspergillus fumigatus* [14,15]. Sepsis-associated mortality in patients suffering from an invasive fungal infection is known to be high, with up to 42% of mortalities observed due to *Candida* spp. and even higher numbers in response to *Aspergillus* spp. infections [16,17,18]. Especially in patients suffering from fungemia, diagnostic weaknesses may substantially contribute to this alarming mortality. Only a small proportion of affected patients show positive blood cultures, and fungal growth on culture media is known to be very slow. Accordingly, several studies have shown that invasive fungal infections are the most frequently missed diagnoses in critically ill patients [19,20,21]. Consequently the administration of life-saving antifungal therapy may either be completely absent, or initiated with a minimum delay of 2–3 days [22]. Indeed, such a delay is known to be associated with an increased mortality [23,24]. On the other hand, molecular biological methods for the detection of fungal isolates (e.g., polymerase chain reaction (PCR)-based methods) have already been shown to be of value (saving time, good sensitivity, and specificity) [25]. However, up to now there are still no commercial assays available [26].

The aims of this observational, prospective clinical study were therefore to (1) assess the incidence, risk factors and relevance with regard to the outcome of fungal infections in patients with septic shock; (2) evaluate the additional benefit of a next-generation sequencing (NGS)-based diagnostic procedure; and (3) assess the host response upon fungal infection by investigating the diagnostic value of β-d-glucan (BG), galactomannan (GM), interferon gamma (INF-γ), tumor necrosis factor alpha (TNF-α), interleukins (IL)-2, -4, -6, -10, -17A, and mid-regional proadrenomedullin (MR-proADM) in septic patients as well as by analyzing the overall host response by expression profiling of *Candida* spp.-infected epithelia in vitro.

## 2. Results

### 2.1. Patient’s Characteristics

In total, 50 patients with septic shock were included in the presented investigation. Patients’ characteristics are presented in Table 1. The underlying septic focus was the abdomen (*n* = 43; 86%), followed by the lung (*n* = 6; 12%), and the urogenital tract (*n* = 1; 2%). The overall 28 and 90 day mortality rates were 22% (*n* = 11) and 34% (*n* = 17), respectively. The median length of intensive care unit (ICU) and hospital stay was 20 and 44 days, respectively.

### 2.2. Fungal Pathogens and Infection Sites

#### Culture-Based Microbiological Diagnostics

**Figure 1:**
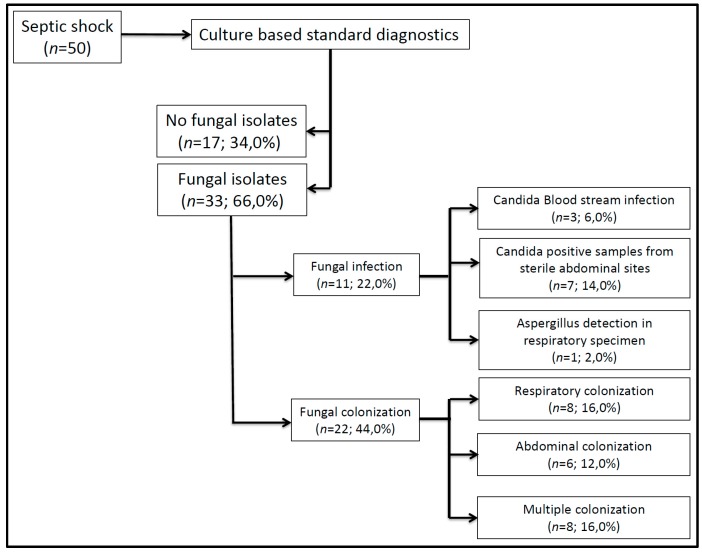
Identification of fungal pathogens in patients with septic shock (*n* = 50).

As assessed by culture-based microbiological diagnostics, fungal pathogens were present in 33 patients (66.0%), whereas 17 patients (34.0%) revealed negative fungal cultures (Figure 1). Fungal isolates were found in single or multiple locations in 25 (75.8%), or 8 (24.2%) patients respectively, and were located at the following sites: respiratory tract (*n* = 17; 51.5%), abdominal site (*n* = 21; 63.6%) and blood culture (*n* = 3; 9.1%) (double-naming feasible, Figure 1). Characteristics of patients with or without fungal pathogens are presented in Table 1. Patients with fungal pathogens more frequently underwent liver surgery prior to study inclusion and the need for renal replacement therapy was shown to be significantly increased. Concerning further markers for morbidity, fungal-positive patients revealed a significantly prolonged duration of mechanical ventilation, and the need for tracheostomy tended to be increased. Moreover, the length of both ICU and hospital stay was significantly prolonged in patients with fungal pathogens. Surprisingly, 28 day mortality was significantly increased in patients without fungal pathogens, whereas 90 day mortality was shown to be comparable.

Based on the group definitions as described in the methods section, fungal colonization and fungal infection was found in 22 (44.0%) and 11 (22.0%) patients, respectively (Figure 1). In colonized patients, 8 (16.0%) participants exclusively revealed *Candida* spp. in respiratory secretions (5× *C. albicans*, 1× *C. albicans* and *C. glabrata*, 2× *C. albicans* and *C.* spp.), whereas in 6 (12.0%) patients *Candida* spp. could only be cultured from drainage fluids (3× *C. albicans*, 2× *C. glabrata*, 1× *C. albicans* and *C*. *glabrata*). In contrast, 8 (16.0%) patients were colonized at both sides (4× *C. albicans*, 1× *C. albicans* and *C.* spp., 3× *C. albicans* and *C. glabrata*). In infected patients, fungemia was found in 3 (6.0%) patients (2× *C. albicans*, 1× *C. glabrata*) and positive abdominal wound swabs were found in 7 (14.0%) patients (4× *C. albicans*, 1× *C. glabrata*, 1× *C. krusei*, 1× *C. albicans* and *C. glabrata*). Moreover, in one (2.0%) patient *Aspergillus fumigatus* was isolated in respiratory tract secretions. Detailed characteristics of patients without a fungal infection (colonized patients as well as patients without any fungal isolates), patients with a fungal colonization, and patients with a fungal infection are presented in Table 2 and Table 3. Concerning risk factors, liver surgery prior to study inclusion as well as liver cirrhosis could be observed more frequently in patients with a fungal infection. Moreover, the duration of ICU stay and mechanical ventilation was significantly prolonged and the need for tracheotomy was significantly increased in patients suffering from a fungal infection (Table 3). Although morbidity was shown to be increased, mortality at 28 and 90 days did not differ significantly between patients with and without a fungal infection.

### 2.3. NGS-Based Microbiological Diagnostics

An unbiased approach for the diagnoses of bloodstream infections based on high-throughput sequencing of cell-free plasma DNA and the calculation of a sepsis indicating quantifier (SIQ)-score has been described previously [27]. Using this approach, bacterial or viral bloodstream infections can be identified with a high sensitivity. For the detection of fungal species in plasma from this cohort, we calculated a modified fungal SIQ score as a product of abundance and significance in each of the patients, which was then compared with clinical microbiology data as well as supplemented data on anti-infective therapy over a trial period of 28 days. Accordingly, we consequently present detailed data of three selected patients, describing the suitability of a NGS-based approach for the detection of invasive mycoses for three different scenarios:

#### 2.3.1. Confirmation of Culture-Based Diagnostics of Candidemia by Fungal SIQ Score

**Figure 2:**
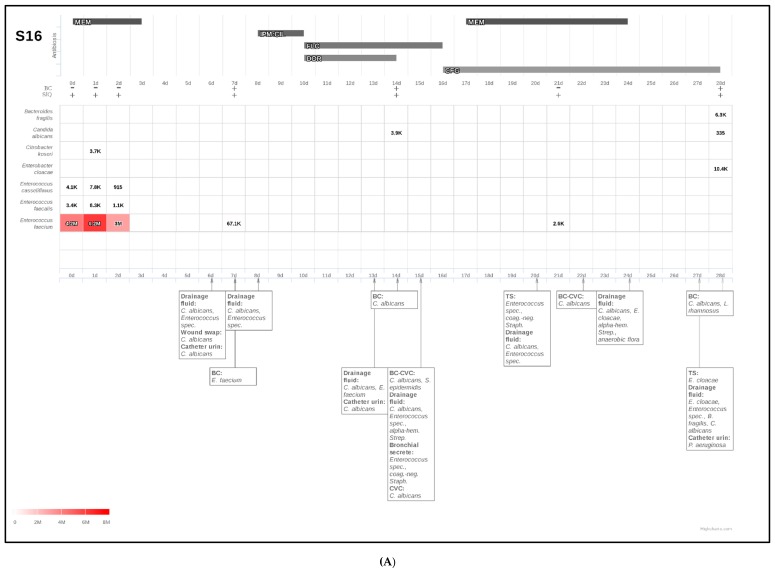

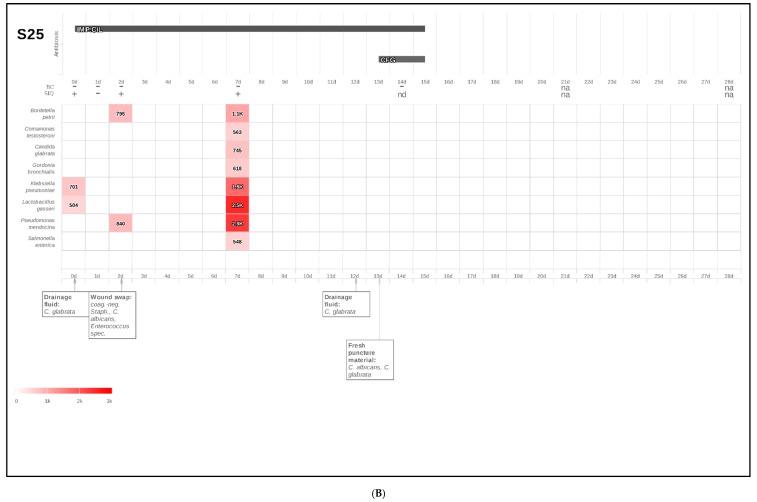

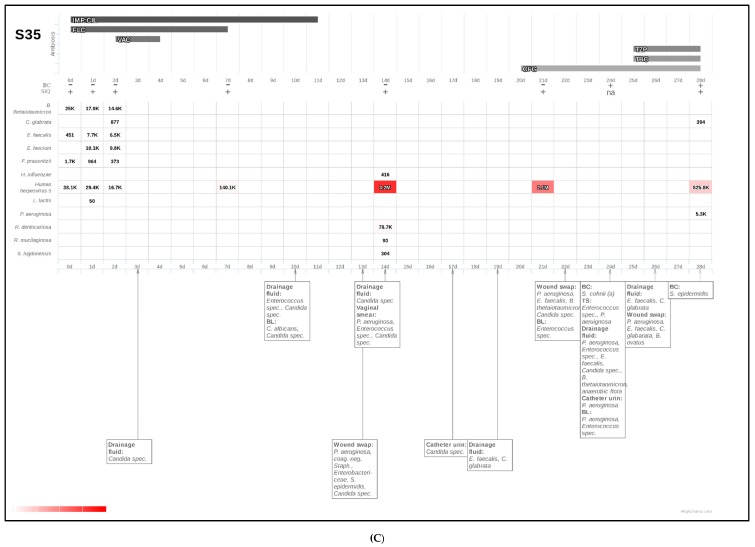
Time course (fungal) SIQ analyses compared with conventional clinical microbiology data of septic patients. The anti-infective treatment regime and (fungal) SIQ scores for species identified via NGS of the respective plasma samples are reported for a time course of 28 days (indicated by the *x*-axis) for patients S16 (**A**), S25 (**B**), and S35 (**C**). Only species identified by SIQ-score analyses are indicated at the left side. Red colored boxes reveal ranking of highest SIQ scores for the respective species in every patient. Pertinent (clinical microbiology) laboratory results are marked using arrows to indicate the day the clinical specimen was obtained. (**A**) A 73-year old male patient presented with a tumor of his bile duct with the need for a palliative resection. The surgical procedure included resections of the bile duct as well as the gallbladder and was followed by a double bypass procedure (biliodigestive anastomosis and gastrojejunal anastomosis). Four days after the initial operation the patient suffered from septic shock due to a duodenal ulcer perforation with the need for a total pancreatectomy. Shortly after, the patient suffered from another small bowel leakage, so that an additional small bowel resection had to be performed. Blood cultures at sepsis onset were shown to be negative, and meropenem (MEM) was administered in terms of an empiric antibiotic therapy. However, the patient suffered from a therapy-refractory course of the disease and *C. albicans* could be isolated from abdominal drainage fluids 6 days after sepsis onset. Accordingly, an additional antifungal treatment with fluconazole (FLC) was initiated. Due to the development of candidemia at 14 days after sepsis onset, this antifungal treatment regime was secondarily escalated towards caspofungin (CFG). These findings were in good agreement with next generation sequencing (NGS) diagnostics in plasma, since the SIQ-score was positive for *C. albicans* at the same timepoint. Abbreviations: NGS, next generation sequencing; SIQ, sepsis indicating quantifier; MEM, meropenem; IPM:CIL, imipenem/cilastatin; FLC, fluconazole; DOR, doripenem; CFG, caspofungin; BC, blood culture; CVC, central venous catheter; TS, tracheal secretion; (**B**) A 65-year old male patient suffered from a Klatskin tumor with the need for a right-sided hemihepatectomy. Due to an abscess at the resection site, the patient suffered from septic shock with the need for an interventional drainage 22 days after the initial operation. The further course was complicated by the development of a right-sided pleural empyema as well as recurrent intra-abdominal abscesses, which were both treated with repeated placements of interventional drainages. Empiric antibiotic therapy at sepsis onset included imipenem/cilastatin (IMP:CIL) in terms of a monotherapy. Culture-based microbiological diagnostics revealed no bacterial growth, whereas *C. glabrata* could be detected in both fluids of already positioned drainages as well as fresh puncture materials respectively. Based on these microbiological findings, the patient was classified as infected, so that an administration of caspofungin was started at 14 days after sepsis onset. Blood cultures remained negative for fungi at all time points. Contrariwise, a next generation sequencing (NGS)-based diagnostic approach in plasma samples of septic patients was able to support the presence of an invasive fungal infection already at 7 days after sepsis onset, since the SIQ-score was shown to be positive for *C. glabrata* at this time point. Unfortunately, a further evaluation of the patient’s course of the disease beyond 14 days after sepsis onset was not possible, since the patient denied further participation in the study. Abbreviations: CFG, caspofungin; IMP:CIL, imipenem/cilastatin; na, not available; nd, not detectable; NGS, next generation sequencing; SIQ, sepsis indicating; (**C**) A 71-year-old female patient presented with a right pleural empyema caused by a liver abscess with the need for a video-assisted thoracoscopy (VATS). One day after VATS, the patient suffered from an acute abdomen with septic shock due to a perforation of the sigmoid colon, so that a removal of the sigmoid colon had to be performed. A second explorative laparotomy was necessary at 10 days after sepsis onset, due to a messy drainage fluid with a suspicion of another bowel leakage. However, during the revision surgery no clear focus could be found. Empiric anti-infective treatment consisted of imipenem/cilastatin (IMP:CIL) in combination with fluconazole (FLC), which was further supplemented by vancomycin (VAC) for 2 days in the early phase after sepsis onset. Anti-infective treatment was stepwise deescalated, so that the patient was free of any antibiotics or antimycotics at 12 days after sepsis onset. In the further course of the disease, the administration of caspofungin (CFG) was started at 20 days after sepsis onset, since the patient did not recover well and drainage fluids were shown to be positive for *Candida* spp. repeatedly starting from 3 days after sepsis onset. In parallel, next generation sequencing (NGS)-based diagnostics revealed a positive SIQ-score for *C. glabrata* also at 3 days after sepsis onset, whereas blood cultures were found to be negative for fungi throughout the whole observation period. The end of the 28 day-observation period was further characterized by an insufficiency of the stump by Hartmann as well as the development of severe pneumonia with the key bacteria Pseudomonas aeruginosa and Enterococcus faecalis, so that another antibiotic treatment phase with piperacillin/tazobactam as well as inhaled tobramycin was initiated. Abbreviations: BC, blood culture; BL, bronchoalveolar lavage; CFG, caspofungin; FLC, fluconazole; IMP:CIL, imipenem/cilastatin; n.a, not available; NGS, next generation sequencing; SIQ, sepsis indicating quantifier; TBC, inhaled tobramycine, TS, tracheal secretion; TZP, piperacilline/tazobactam; VAC, vancomycin.

Patient S16 suffered from candidemia (as assessed by culture-based diagnostics) fourteen days after sepsis onset due to recurrent small bowel leakage. However, *Candida* spp. were present in drainage fluids already one week prior to the onset of candidemia (at six days after sepsis onset), which led to the initiation of a fluconazole therapy and was secondarily switched to caspofungin, as soon as candidemia occurred (Figure 2A). Blood cultures revealed positive results for *C. albicans* 2 and 4 weeks after inclusion into the study which was confirmed by SIQ score analyses (Figure 2A) However, high-throughput sequencing already revealed low levels of *C. albicans* sequence reads at the day of inclusion, but were not considered significant under the stringent criteria applied for this analyses (material not intended for publication). Nevertheless, with an increased sensitivity this NGS approach holds promise to diagnose blood stream infection significantly earlier than conventional microbiological culturing.

#### 2.3.2. NGS-Based Diagnosis of Candidemia in Patients with Suggested Infection Based on Microbiological Identification of *Candida* spp. in Other Than Blood Culture

Patient S25 suffered from recurrent intra-abdominal abscesses as well as a right-sided pleural empyema following a right-sided hemihepatectomy due to a Klatskin tumor. *Candida glabrata* and *Candida albicans* were found by microbiological testing of drainage fluids, wound swabs, as well as fresh puncture materials (Figure 2B). Although blood cultures remained negative for fungi at all time points, NGS-based calculation of a fungal SIQ score was positive (at 7 days after sepsis onset) for *Candida glabrata* already 6 days before microbiological testing of fresh puncture materials (at 13 days after sepsis onset) and therefore strongly supported the earlier presence of a candidemic infection. In this context, the identification of *C. glabrata* infection was further supported by positive drainage fluid cultures.

#### 2.3.3. Detection of Invasive Mycoses by Fungal SIQ Score Even in Those Patients Which Have So Far Been Classified as Colonized Based on Culture-Based Diagnostic Procedures

Due to restrictions including the limited sensitivity of culture-based diagnostics, some patients might be falsely classified as colonized despite the presence of an invasive fungal infection. Accordingly, a more sensitive and reliable diagnostic procedure would be of value in order to minimize false-negative test results. For example, patient S35 (Figure 2C) was classified as being colonized (as assessed by culture-based diagnostics in drainage fluids or wound swaps), although calculation of the SIQ score clearly revealed signs of an invasive *Candida glabrata* infection already at 2 days after sepsis onset. In contrast, the identification of *C. glabrata* at the species level was provided by microbiological diagnostics 17 days later in drainage fluid, which might contribute to the delayed prescription of caspofungin in that patient. Knowledge of these NGS-based results would probably have led to the initiation of an antifungal therapy, which might have been of relevance for the presented patient.

### 2.4. Antifungal Therapy

In total, 21 of 50 (42.0%) patients received an antifungal therapy during study participation. Of the 17 patients without any fungal isolates, 2 (11.8%) patients received an empiric antifungal therapy. Of the remaining 33 patients with fungal isolates, 19 (57.6%) patients received an antifungal therapy, which was initiated in terms a specific therapy in 15 (78.9%) patients. Conversely, treatment was initiated in terms of an empiric therapy in the remaining 4 (21.1%) cases, which was stopped later on in all of these patients. In 7 (33.3%) patients, the initial antifungal therapy was changed during the course of the disease. Antifungals used for the first and second line therapy are reported in Table 4.

### 2.5. (1,3)-β-d-Glucan (BG)

**Figure 3:**
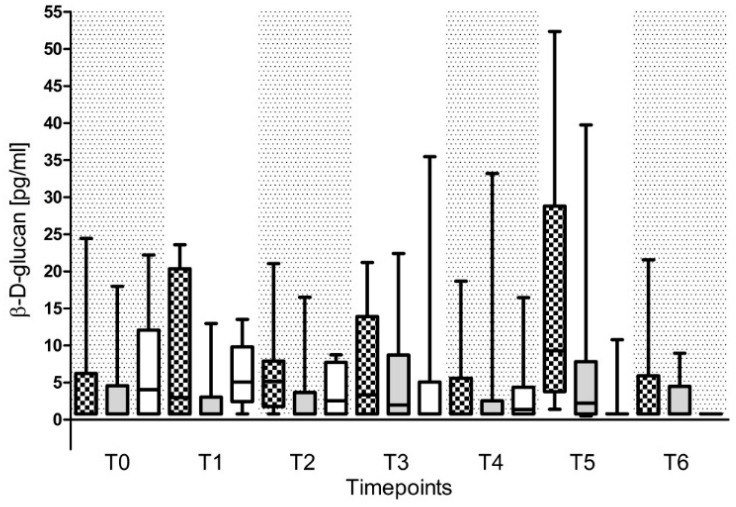
Plasma concentrations of β-d-glucan (BG) in patients with septic shock. Plasma concentrations of BG were measured in patients suffering from septic shock with a fungal infection (grey squared box), a fungal colonization (grey plane box) or without any fungal findings (white box). Plasma samples were collected at the onset of septic shock (T0), and 1 day (T1), 2 days (T2), 7 days (T3), 14 days (T4), 21 days (T5), and 28 days (T6) afterwards. Data in box plots are given as median, 25th percentile, 75th percentile with the 10th as well as 90th percentile at the end of the whiskers.

Plasma concentrations of BG were comparable between the three subgroups throughout the entire study period (Figure 3) and therefore failed to be of diagnostic value for the prediction of a fungal infection. Even in patients suffering from candidemia, plasma concentrations of BG were not increased reliably.

### 2.6. Galactomannan (GM)

Plasma concentrations of GM remained below the cut-off value of <0.5 in 46 of 50 patients (92.0%). Conversely, 4 patients (8.0%) presented with sporadically increased plasma concentrations of GM above the cut-off value without any other (clinical, radiological, cultural) signs or risk factors for an IA (material not intended for publication). In these cases, increased plasma concentrations of GM were most probably attributable to the underlying antibiotic therapy (e.g., piperacillin-tazobactam), which is well known to be associated with increased GM concentrations [28,29].

One patient presented with the diagnosis of an IA as assessed by cultural detection of *Aspergillus fumigatus* in BALF, which was confirmed by high-resolution computed tomography. Moreover, GM concentrations in BALF were increased above the cut-off value, whereas plasma concentrations of GM remained below the cut-off value at all time points. Apart from septic shock as well as pre-existing adipositas per magna and insulin-depending diabetes mellitus, the patient did not suffer from classical predisposing risk factors for IA (e.g., neutropenia, hemato-oncological diseases treated with cytotoxic agents, intake of corticosteroids, innate or acquired immunodeficiency). The patient was treated with liposomal amphotericin B for 6 weeks, which led to a decrease of GM in BALF below the cut-off value. Moreover, culture of BALF remained negative for *Aspergillus fumigatus* after the end of the treatment period.

### 2.7. Anti-Candida Antibody Titer

In the subgroup of patients without any fungal findings (*n* = 17), 4 patients (23.5%) presented with a “false” positive anti-*Candida* antibody titer (>1:320), whereas colonized patients (*n* = 22) were shown to have positive test results in 81.8% (*n* = 18) of cases. Patients suffering from a fungal infection (*n* = 11) also revealed positive test results in 81.8% (*n* = 9) of cases, but unfortunately two patients presenting with candidemia (at sepsis onset) failed to show a positive anti-*Candida* antibody titer.

### 2.8. Inflammation as Well as Infection Marker Levels

**Figure 4:**
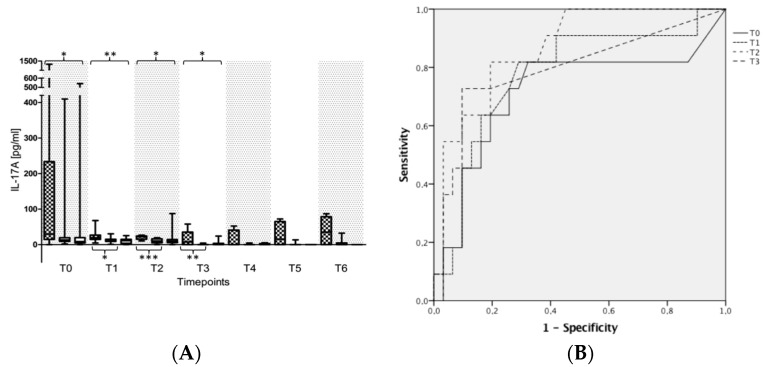
Plasma concentrations of interleukin (IL)-17A in patients with septic shock. Legend: (**A**) Plasma concentrations of IL-17A were measured in patients suffering from septic shock with a fungal infection (grey squared box), a fungal colonization (grey plane box) or without any fungal findings (white box). Plasma samples were collected at the onset of septic shock (T0), and 1 day (T1), 2 days (T2), 7 days (T3), 14 days (T4), 21 days (T5), and 28 days (T6) afterwards. Data in box plots are given as median, 25th percentile, 75th percentile with the 10th as well as 90th percentile at the end of the whiskers. Concerning symbolism and higher orders of significance: * *p* < 0.05, ** *p* < 0.01, *** *p* < 0.001; (**B**) Receiver operating characteristic (ROC) analysis with IL-17A in all participating patients at sepsis onset (T0), and 1 day (T1), 2 days (T2) as well as 7 days (T3) afterwards with regard to the prediction of a fungal infection up to day 28. Patients suffering from a fungal infection represented the target group, whereas both, patients with a fungal colonization as well as patients without any fungal isolates served as controls for this ROC-analysis.

**Figure 5:**
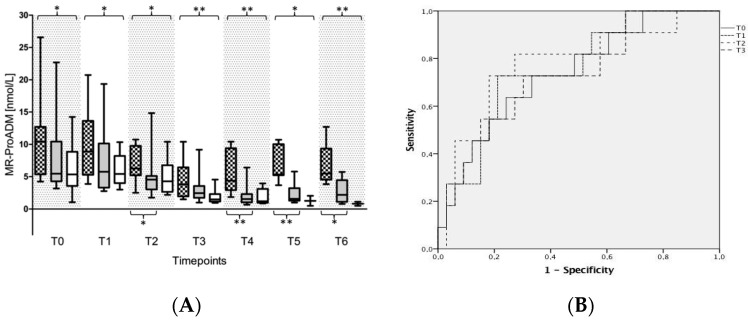
Plasma concentrations of mid-regional proadrenomedullin (MR-proADM) in patients with septic shock. Legend: (**A**) Plasma concentrations of MR-proADM were measured in patients suffering from septic shock with a fungal infection (grey squared box), a fungal colonization (grey plane box) or without any fungal findings (white box). Plasma samples were collected at the onset of septic shock (T0), and 1 day (T1), 2 days (T2), 7 days (T3), 14 days (T4), 21 days (T5), and 28 days (T6) afterwards. Data in box plots are given as median, 25th percentile, 75th percentile with the 10th as well as 90th percentile at the end of the whiskers. Concerning symbolism and higher orders of significance: * *p* < 0.05, ** *p* < 0.01; (**B**) Receiver operating characteristic (ROC) analysis with MR-proADM in all participating patients at sepsis onset (T0), and 1 day (T1), 2 days (T2) as well as 7 days (T3) afterwards with regard to the prediction of a fungal infection up to day 28. Patients suffering from a fungal infection represented the target group, whereas both, patients with a fungal colonization as well as patients without any fungal isolates, served as controls for this ROC-analysis.

Plasma levels of acute phase proteins (such as C-reactive protein or procalcitonin), leukocytes as well as general inflammation marker levels (such as IL-2, TNF-α) in patients without any fungal isolates, suffering from a fungal colonization or a fungal infection are presented in Table S1. With regard to fungal immunity, special attention should be given to the plasma levels of INF-γ, IL-4, -6, -10, -17 as well as MR-proADM. Plasma levels of the pro-inflammatory cytokine INF-γ were shown to be significantly elevated in patients suffering from a fungal infection in comparison to both control groups, starting from seven days after sepsis onset (T3). This increase in INF-γ was paralleled by a significant release of the immunosuppressive cytokines IL-10 and -4 in infected patients (Table S1). Plasma levels of IL-6 were shown to be significantly elevated in patients suffering from a fungal infection in comparison to septic patients with a fungal colonization or without any fungal findings at different time points especially in the early course of the disease (e.g., at T0, T1) (Table S1). In parallel, IL-17A was also shown to be significantly increased in septic patients suffering from a fungal infection in comparison to septic patients with a fungal colonization or without any fungal findings within the first 7 days after sepsis onset (Figure 4A). Therefore, IL-17A was found to be a suitable tool for early identification of patients with a fungal infection as assessed by receiver operating characteristic (ROC)-analyses (ROC-area under the curve (AUC) for patients with a fungal infection vs. non-infected patients (i.e., patients without any fungal isolates + colonized patients) e.g., at T0: 0.714; Cut-Off 14.165 pg/mL → Sens. 0.818; 1-Spec. 0.323; T1: 0.776; Cut-Off: 14.22 pg/mL → Sens. 0.818; 1-Spec. 0.29; T2: 0.865 Cut-Off 15.00pg/mL → Sens. 0.818; 1-Spec. 0.194, etc.) (Figure 4B). The same holds true for plasma levels of MR-proADM, which were shown to be significantly increased in infected patients in comparison to both colonized patients and those without any fungal findings (Figure 5A). Accordingly, MR-proADM was also shown to be a suitable tool for the identification of patients with a fungal infection as assessed by a receiver operating characteristic (ROC)-analyses (ROC-area under the curve (AUC) for patients with a fungal infection vs. non-infected patients (=see above) e.g., at T0: 0.738; Cut-Off: 6.99 nmol/L → Sens. 0.727; 1-Spec. 0.333; T1: 0.755; Cut-Off: 8.53 nmol/L → Sens. 0.727; 1-Spec. 0.212; T2: 0.774; Cut-Off 5.10 nmol/L → Sens. 0.818; 1-Spec. 0.273, etc.) (Figure 5B).

### 2.9. Conserved Host-Response Patterns during Epithelial Infections with Candida spp.

**Figure 6:**
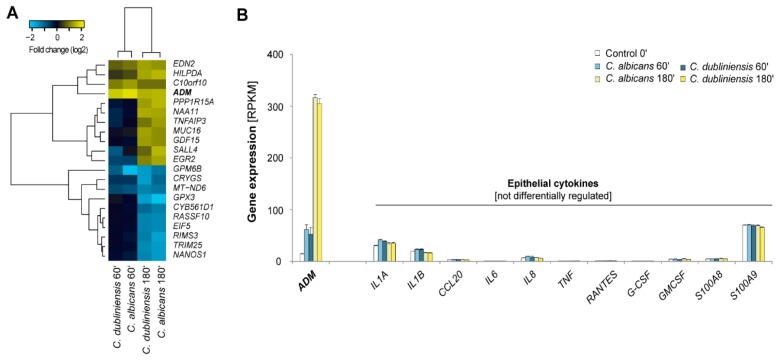
Early transcriptional host response of vulvovaginal RHE. Legend: (**A**) Hierarchical clustering of the set of 21 differentially expressed genes based on their fold changes. For each infected condition the uninfected control at the corresponding timepoint dealt as reference condition; (**B**) Expression values of late-stage *Candida*-induced cytokines compared to ADM.

In order to evaluate the early response to a fungal colonization or infection of the host, vulvovaginal reconstituted human epithelia (RHE) were infected with two different fungal species from the *Candida* clade with an MOI of 10:1—the most prevalent member *C. albicans* and a clinically rather less important agent *C. dubliniensis*. Upon adhesion, *C. albicans* immediately started forming hyphae and penetrating the epithelia while *C. dubliniensis* remained in yeast form mainly adhering but not invading (material not intended for publication). In this context it was remarkable that the transcriptional response patterns were highly conserved between the two applied *Candida* species within the investigated initial three hours (Figure 6A, Table S2). Beyond that, the observed responses are very specific with only 21 detected genes being differentially regulated. Indeed, among the three rapidly induced genes (after 60 min of infection) we found that ADM had the strongest transcriptional activation with both *Candida* spp. For comparison, we evaluated the expression levels of cytokines which have been associated with *C. albicans* invasion after 24 h [30] but we did not observe any induction at this very early infection stage (Figure 6B). Thus, the products of *ADM* are likely to be a far earlier diagnostic marker for fungal infections on epithelia than e. g. IL-1A/B or IL-8. Interferon gamma (INF-γ), tumor necrosis factor alpha (TNF-α), as well as interleukins (IL)-2, -4, -6, -10, and -17A are not expressed at all in the epithelium.

## 3. Discussion

Within this combined clinical and experimental investigation, an NGS-based diagnostic approach and plasmatic measurements of MR-proADM and/or IL-17A were identified to be suitable for a comprehensive, reliable, and fast diagnosis of mycoses in patients suffering from septic shock. Moreover, ADM appeared to be one key element of various conserved host response patterns during epithelial infections with *Candida* spp.; however its functional characterization needs to be determined in further experimental studies.

### 3.1. Invasive Fungal Infections—A Clinical Dilemma

In patients suffering from severe sepsis or septic shock, positive blood cultures are obtained in only one third of cases despite a proven underlying bacterial infection [31,32,33]. This is partially attributable to technical shortfalls in blood culture acquisition, but is also due to very low rates of viable microorganisms or fastidious organisms in the blood stream [34]. Especially culture-based diagnostics for the detection of fungemia are known to be associated with relevant weaknesses [35]. Although a fungal infection might be present in up to 30% of patients with severe sepsis or septic shock, only 2–3% of these infections can be proven by blood culture [31]. Accordingly, a molecular approach with higher sensitivity for sepsis might, therefore, overcome the aforementioned limitations of classic microbiological diagnostics. However, state of the art molecular approaches based on PCR amplification of target sequences suffer from limited power to discriminate between contaminations, colonization, and infections, often revealing ambiguous or invalid results. Moreover, up to now there are still no commercial assays available for the identification of fungal isolates [26]. The present clinical investigation therefore aimed to assess the incidence, risk factors and outcome relevance of fungal infections in patients with septic shock and also tried to evaluate the additional benefit of a next-generation sequencing (NGS)-based diagnostic procedure in affected patients.

According to the Candida-Score of Leon et al. [36] (Table S3) and in line with the results of Leroy et al. [37], patients participating in the present clinical investigation were identified to be at high risk of a fungal infection. In line with the recent literature, fungal pathogens were found in our cohort in two thirds of all septic patients, whereas patients with fungal isolates were shown to be either colonized in 66.6% or suffered from an invasive infection in 33.3% [14]. Resulting fungal isolates were also comparable to previous epidemiologic studies, in which *Candida* spp. (*C. albicans* and *C. glabatra*) were isolated most frequently [14,31,38,39]. Moreover, the incidence of invasive pulmonary Aspergillosis (caused by *Aspergillus fumigates*) was comparable to previously published investigations [10,14,40,41].

All patients suffered from septic shock and therefore need to be considered as critically ill, as assessed by various disease severity scores (e.g., APACHE II, SOFA and SAPS II). Although these scores did not differ significantly between patients with or without fungal isolates as previously described by Lichtenstern et al. [14], the SOFA-score was shown to be significantly increased in patients suffering from a fungal infection in comparison to patients with a fungal colonization (Table 2) as well as patients without a fungal infection (Table 3). Accordingly, disease severity and the presence/occurrence of fungal infections seem to be closely related. The same holds true for the requirement of renal replacement therapy, mechanical ventilation as well as tracheostomy, which were all significantly increased in either patients with fungal isolates in comparison to those without fungal isolates (Table 1), or in patients with a fungal infection in comparison to patients with a fungal colonization (Table 2), respectively patients without a fungal infection (Table 3) [42,43].

Within the presented investigation, the overall mortality was 22% and 34% at 28 and 90 days respectively. Although this mortality is substantially lower than the APACHE II predicted mortality of 55–75% [44], the presented mortality data are in line with a previously published investigation of our workgroup [14]. Moreover, mortality of patients suffering from a fungal infection within the presented investigation is in line with the current literature, reporting mortality rates of 30–70% due to aspergillosis and of 20–70% due to candidiasis [14,45,46,47,48]. Surprisingly, the presence of fungal isolates, with respect to a fungal infection, did not result in a deterioration of the patient’s outcome (as assessed by the 28 or 90 day mortality) within the presented investigation as previously described by Montravers et al. [49] or Lichtenstern et al. [14], which might probably be attributable to the small cohort of patients included within the recent investigation. However, in line with the current literature, the length of ICU as well as hospital stay were significantly prolonged and the rate of fascia dehiscences was significantly increased in patients showing fungal isolates or suffering from a fungal infection [49] in comparison to the different peer groups (Table 1, Table 2 and Table 3). In line with the results of Fiore et al. and Theocharidou et al., special attention should also be placed on the role of the liver in the context of mycoses, since pre-existing liver cirrhosis as well as liver surgery prior to sepsis onset have been closely related to the occurrence of a fungal infection [50,51].

### 3.2. Solutions to the Fungal Dilemma

As described above, fungi are common pathogens in critically ill patients and the presence of a fungal infection is associated with an increase in morbidity in comparison to colonized as well as non-infected patients. Accordingly, a fungal infection needs to be detected as early as possible in order to initiate a targeted life-saving antifungal therapy, since delayed initiation of appropriate antifungal therapy has been shown to increase morbidity and mortality [23,24,52,53]. However, culture-based diagnostic procedures for the detection of invasive fungal infections (e.g., candidemia) are associated with relevant weaknesses [54], so that molecular biological methods for the detection of the causative fungal pathogen might be of additional value. Within this context, PCR-based methods, in particular, have been examined during the last years [25]; however, the occurrence of ambiguous results as well as the limitations in the quantitative measurement of the pathogen load in patients’ samples, and the detection of resistance markers, are known limitations of these PCR-based diagnostic approaches. Accordingly, new solutions to this fungal dilemma need to be developed.

### 3.3. Next-Generation Sequencing (NGS) Diagnostics of Fungal Infections

The concept of our work was therefore to develop an alternative diagnostic platform for the identification of fungal pathogens based on unbiased sequence analyses of circulating cell-free DNA (cfDNA) by NGS from plasma samples of septic patients, which are at high risk for a fungal infection. In contrast to PCR-based technologies, this NGS-based diagnostic platform is an open platform, therefore providing the opportunity to detect bacterial, viral as well as fungal pathogens in a single assay. Moreover, this NGS-based approach is quantitative, unbiased and allows for the discrimination of signal reads from noise (caused by contaminant or commensal species) via SIQ score calculation. Briefly, by comparing abundances of species reads from patients with those from controls considered not to be infected one can calculate a statistical measure for the expected occurrence of reads for every species. Contaminating or commensal species reveal a species-specific background level which is statistically distinguishable from infecting levels. A comparable NGS-based diagnostic approach for early diagnostics of bacteremia has (among others) recently been published by our workgroup [27,55,56]. However, exact data on the detection of fungal pathogens in the plasma of patients at high risk of a fungal infection are still lacking, although a specific protocol for the use of NGS for the detection of fungal isolates has recently been published by our workgroup [57]. By the use of this newly designed “fungal SIQ score” in plasma samples of patients with septic shock, within the present investigation we were able to (1) confirm culture-based diagnostics of candidemia; (2) support culture-based diagnostics of invasive fungal infections in non-candidemic patients; and (3) detect invasive mycoses even in those patients, which have so far been classified as colonized based on culture-based diagnostic procedures. Moreover, the NGS-based approach revealed signs of an invasive fungal infection already prior to the first culture-based findings, thus providing the opportunity to initiate a targeted life-saving antifungal therapy in infected patients much earlier than previously possible.

Accordingly, this approach represents a promising diagnostic platform for the diagnosis of cases where classic microbiological or molecular diagnostic approaches fail. This holds true especially for critically ill patients at high risk of developing fungal infections or suffering from a bloodstream infection with fungi. However, although we think that this NGS-based approach appears to be more sensitive and specific than state-of-the-art technologies, additional clinical trials are needed in order to exactly define the sensitivity and specificity as well as the positive and negative predictive value, as this case study was limited by the low number of patients. Up to the time when such new diagnostic techniques will be available in clinical routine, the clinician is restricted to classic microbiological diagnostic approaches with their specific limitations, which is made more difficult by non-specific clinical manifestations as well as delayed radiologic findings.

### 3.4. Fungal Biomarkers for the Diagnosis of Fungal Infections

Nowadays, especially the use of non-invasive diagnostic tools such as BG or GM is aimed at attenuating the above mentioned inefficiencies in the diagnosis of invasive fungal infections, although these biomarkers are also far from perfect. Several studies have addressed the potential usefulness of BG and GM for the diagnosis of an invasive fungal infection in neutropenic as well as non-neutropenic patients [47,58,59,60]. Accordingly, the potential value of both biomarkers has been acknowledged by their inclusion in the revised European Organisation for Research and Treatment of Cancer/National Institute of Allergy and Infectious Disease Mycoses Study Group (EORTC/MSG) criteria definitions of invasive fungal diseases/infections [61].

BG is a common cell wall component of various medically important fungi, including *Candida* spp. and *Aspergillus* spp. Although plasmatic BG measurements can easily be performed by the use of commercially available assays, reports on the screening performance of BG are scarce, the test performance varies, and published data on BG are hallmarked by relevant limitations. Nevertheless, recent meta-analyses on the diagnostic value of plasmatic BG for the detection of patients with invasive fungal diseases suggest that the BG assay is a useful screening tool for the discrimination of patients with or without an invasive fungal infection [62,63,64]. However, since the BG assay is not absolutely sensitive and specific for an invasive fungal infection, BG results should always be evaluated together with clinical and microbiological findings [63,64]. Accordingly, the workgroups of Digby et al. and Marty et al. were able to reveal false positive BG plasma levels in patients suffering from a bacterial infection [65] or receiving an antibiotic treatment [66]. These diagnostic deficiencies also became evident within the present investigation, since plasma levels of BG did not differ significantly between patients suffering from a fungal infection and colonized patients, or patients without any fungal isolates.

GM is a heat-stable polysaccharide present in the fungal wall of most *Aspergillus* spp., a saprophytic filamentous fungus that can usually be isolated in the environment [67]. In the course of an invasive disease, GM can be detected in different body fluids (e.g., serum, bronchoalveolar lavage fluid (BALF), cerebrospinal fluid). Most frequently, invasive Aspergillosis (IA) is an infection of the lower respiratory tract and primarily affects immunocompromised patients suffering from prolonged neutropenia or following hematopoietic organ transplants [68]. However, IA has nowadays been recognized as an emerging infectious disease also in critically ill ICU patients in the absence of traditional IA risk factors (as described above, e.g. severe neutropenia >10 days, allogenic stem-cell transplant recipient, prolonged corticosteroids use, intake of T-cell immunosuppressants, inherited severe immunodeficiency) [61,69,70,71,72]. Rather, chronic obstructive pulmonary disease (COPD), short-term corticosteroid use, acute respiratory distress syndrome (ARDS), severe sepsis/septic shock, H1N1 virus infection, decompensated chronic liver disease and acute renal failure are risk factors for IA in critically ill patients [70,71,72]. Due to the aforementioned diagnostic weaknesses as well as a great heterogeneity in the different study populations, incidence and mortality rates of IA range from 0.02–19%, and from 46–95%, respectively [70,73]. Moreover, in some cases the proof of IA has only been made by post-mortem samplings of critically ill non-survivors, thus clearly underlying a diagnostic gap in the accuracy of recent diagnostic approaches for the detection of IA [19,20,21]. Especially since a positive respiratory culture is present in only 40–50% of affected patients, non-invasive determination of GM in patients’ samples is known to be of value for the diagnosis of invasive Aspergillosis (IA) [74]. Serum measurements of GM in high-risk neutropenic patients reveal a sensitivity as well as specificity of 82% and 81% respectively (cut-off 0.5 optical density index) [75]. However, GM results in non-neutropenic patients are much worse due to a clearance of GM by neutrophils, thus limiting the suitability of GM serum levels for the diagnosis of IA in critically ill patients [76]. Accordingly, either consecutive serum GM determinations [77] or single measurements of GM in BALF [78] were shown to provide a better diagnostic accuracy for the diagnosis of IA, whereas the optimal GM cut-off value in BALF (GM cut-off: 0.5 vs. 0.8 vs. 1.0) is still a matter of debate [78,79,80]. Of note, also the combination of GM with BG is known to increase the specificity value for diagnosing IA. Thus, if clinical conditions are feasible, a combination of both assays is recommended in order to rule in diagnoses for at-risk patients [63].

### 3.5. Immune Monitoring as a Diagnostic Tool in Fungal Infections

The human body is steadily confronted with a myriad of pathogens at its interfaces between inside and outside. Especially *Candida* spp. frequently colonize mucosal surfaces throughout the body, but usually without inducing infection. However, this only works as our body has adapted to this condition by establishing physical barriers (e.g., mucosal and skin) as well as setting guardians to protect these barriers. Innate immunity represents the first line of defense against invading fungi, since neutrophils as well as monocytes/macrophages contribute to phagocytosis and direct killing of *Candida* spp. In more detail, fungal pathogen associated molecular patterns (PAMPs, e.g., β-glucans or mannans) are mainly recognized by two types of pattern recognition receptors (PRRs), Toll-like receptor (TLR)-2 and TLR-4, or C-type leptin receptors (CLRs, mainly Dectin-1) on host cells, which are known to be upregulated in the case of an invasive fungal infection. These innate sensing mechanisms on dendritic cells (DCs), macrophages and epithelial cells ultimately lead to the activation of multiple intracellular pathways with the production of distinct sets of cytokines and other mediators, resulting in the differentiation of CD4^+^ T helper (T_h_) cells as well as regulatory T (T_reg_) cells in response to fungi [81].

A predominance of T_h_1 cells arises from a TLR- as well as CLR-mediated activation of DCs throughout fungal PAMPs. Activation of T_h_1 cells is associated with an enhanced synthesis of the signature cytokine IFN-γ, which is known to be essential for an optimal activation of phagocytes at the site of infection, thus promoting optimal fungal clearance. In contrast, failure of the IFN-γ dependent axis between T_h_1 cells and phagocytes might be a risk factor for overwhelming fungal infections [82]. Accordingly, patients suffering from a fungal infection within the presented investigation were characterized by significantly increased plasma levels of IFN-γ especially at later stages after sepsis onset (Table S1).

Contrariwise, a predominance of T_h_2 cells seems to counteract protective T_h_1 cell responses due to an inhibition of fungal clearance, thus favoring fungal infections or a disease relapse [83,84,85]. IL-4 was shown to be the signature cytokine for T_h_2 cell depending inflammation; whilst in contrast an inhibition of IL-4 dependent pathways resulted in a restoration of antifungal properties [86]. Within the presented investigation leveling of IL-4 was shown to be comparable to IFN-γ (Table S1), thus suggesting an activation of both, T_h_1 and T_h_2 cell-dependent pathways in response to invasive fungal infections.

Apart from T_h_1 and T_h_2 cells, another T-helper cell type (named T_h_17) has recently attracted attention in the context of fungal immunity [87]: These cells are activated by IL-6, IL-23 as well as IL-1ß and are characterized by the ability to synthesize IL-17A, representing the signature cytokine of T_h_17 cells. IL-17A is known to mobilize neutrophils, to activate phagocytosis of neutrophils and macrophages as well as to induce the synthesis of defensins by epithelial cells [81,87,88,89,90]. Accordingly, IL-17 can be considered to be a key mediator of defense against candidiasis and its plasma levels might therefore be used for the diagnosis of patients suffering from an invasive fungal infection. Indeed, Krause and colleagues were recently able to show that candidemic patients have significantly higher IL-17A plasma levels in comparison to non-candidemic patients [91], thus suggesting a potential role of IL-17A for the anticipation of invasive candidiasis. These results can be clearly supported by our investigation, since IL-17A was identified as a promising tool for the identification of patients suffering from a fungal infection. In parallel with significantly elevated plasma levels of IL-17A, IL-6 as the major activating cytokine of T_h_17 cells also revealed significantly increased plasma levels in patients with a fungal colonization, respectively infection (Table S1). However, one has to keep in mind that high IL-17A levels are especially associated with *Candida* spp., whereas *Aspergillus* spp. were shown to be poor inducers of IL-17A [92]. Accordingly, the one patient suffering from IA within the presented investigation also revealed lower plasma levels at the time of diagnosis in comparison to patients suffering from an invasive Candidiasis. In summary, T_h_17 cells seem to be of great importance for the host protection against fungi, and their signature cytokine IL-17A was identified to be a promising biomarker for the identification of patients suffering from an invasive candidiasis. Nevertheless, the release of this cytokine is not specific for fungal immune reactions. Elevated plasma levels of IL-17A may also occur in the context of bacterial sepsis, as described for a cecal ligation and puncture (CLP)-induced polymicrobial sepsis in mice [93]. This may have led to an increase in IL-17A plasma levels even in study patients without a fungal infection, resulting in a relevant overlap between the different subgroups as well as leading to a relevant number of outliers in all subgroups of patients, especially in the early phase after sepsis onset.

In order to keep the balance between an effective elimination of the fungal invader and acceptable collateral tissue damage, another T-helper cell subtype was shown to be of relevance. Via their signature cytokine IL-10, so called regulatory T-cells (T_reg_) are able to dampen the immune response, thus reducing damage to the host but conversely resulting in a state of immunosuppression [81]. This might be due to the fact that IL-10 is known to be an inductor of T_h_2 cells, thus counteracting the pro-inflammatory effects of an IFN-γ driven T_h_1 cell response in fungal immunity. Accordingly, an inverse relationship between IL-10 and IFN-γ was suggested by several investigators [82] that may potentially result in a high susceptibility to fungal infections especially in those patients presenting with high IL-10 levels [94]. However, within the presented investigation of our workgroup, high IL-10 levels could be observed in patients suffering from a fungal colonization or infection in parallel with elevated IFN-γ levels, thus being a consequence, rather than a cause of the underlying fungal infection.

Adrenomedullin (ADM) represents a vasodilatory peptide hormone of 52 amino acids, which is released in several disease states such as heart failure, renal failure, respiratory failure, liver cirrhosis, cancer, as well as infectious diseases incl. sepsis [95]. The synthesis of ADM is widely distributed in several tissues throughout the whole body (kidney, lung, heart, blood vessels, etc.), requiring two consecutive cleaving steps starting from preproadrenomedullin, an initial preprohormone. This preproadrenomedullin is converted into proadrenomedullin by cleavage of the signal peptide, so that three vasoactive peptides (ADM, the aminotemrinal peptide of proadrenomedullin (PAMP), and adrenotensin) as well as a region without known activity, entitled MR-proADM, remain [95]. MR-proADM derives from the final proADM molecule in a ratio of 1:1 with ADM and therefore represents the levels and activity of ADM, which is almost inaccessible for biochemical analysis [95]. Concerning its clinical usefulness, MR-proADM is known to be of diagnostic value for the discrimination of critically ill patients with all-cause sepsis (independent of the underlying microbial pathogen) from those without an infectious stimulus. Within this context, MR-proADM can either be used alone or in combination with other biomarkers (e.g., PCT) [96]. Increased levels of MR-proADM (in parallel to increased ADM levels) were also shown to be associated with disease severity and the magnitude of organ failure, so that MR-proADM has proven to be of prognostic value in septic patients [97]. This prognostic value of MR-proADM was further highlighted by a recently published prospective, observational study by Andaluz-Ojeda et al., in which MR-proADM was the only biomarker to predict sepsis mortality in all severity groups independent of the degree of organ failure (according to the Sequential Organ Failure Assessment (SOFA) score) [98]. Moreover, ADM and PAMP appear to play a critical role in the host defense against systemic infections. Accordingly, expression of ADM is enhanced in connection with a polymicrobial insult, thus reaching adequate mucosal levels in order to serve as a local antimicrobial peptide [99]. Both, ADM and PAMP have shown to be active against Gram-negative and Gram-positive bacteria as well as *Candida albicans* [100,101,102,103,104] and ADM was not only identified as the earliest target gene in response to epithelial infections with *Candida* spp. within our recent experimental investigation but also in a recent study from Kuhbacher and colleagues after 24 h of infection [105]. We thus conclude that (1) MR-proADM represents a key mediator of immunity in the context of candidiasis and (2) plasma level measurements of MR-proADM are therefore a suitable tool for the diagnosis of patients suffering from an invasive fungal infection. This is further supported by the investigation of Angeletti et al., in which MR-proADM levels were shown to be superior to PCT with regard to the identification of patients suffering from Gram-positive sepsis as well as yeast sepsis [96]. Therefore, the routine use of MR-proADM measurements in patients at a high risk of an invasive fungal infection should therefore be taken into account.

### 3.6. Limitations

Although the results of our combined clinical and experimental investigation appear to be sound and conclusive, the following limitations need to be addressed in connection with the presented manuscript. The clinical investigation was performed in terms of an observational single-center study and is therefore characterized by a small number of participating patients, representing a highly selective cohort of critically ill patients suffering from sepsis/septic shock. Moreover, within the experimental part of the investigation, a reconstituted human vulvo vaginal epithelium model was used for the assessment of the host response to infections with *Candida* spp. This experimental cell culture model does not reflect the main fungal infection sites in patients suffering from sepsis, so that the results cannot be transferred without restrictions.

## 4. Materials and Methods

### 4.1. Study Design

The observational clinical study was approved by the local ethics committee (Ethics Committee of the Medical Faculty of Heidelberg, Trial Code No. S-097/2013/German Clinical Trials Register: DRKS00005463) and was conducted in the surgical intensive care unit of Heidelberg University Hospital, Germany, between November 2013 and January 2015. All study patients or their legal designees gave written informed consent. In total 50 patients suffering from septic shock according to the criteria of the Surviving Sepsis Campaign: International Guidelines for Management of Severe Sepsis and Septic Shock 2012 were enrolled in this study [106]. Treatment of patients with septic shock included early-goal directed therapy [107], elimination of the septic focus and broad-spectrum antibiosis [3,107,108]. Blood samples were collected at sepsis onset (T0) and 1 day (T1), 2 days (T2), 7 days (T3) 14 days (T4), 21 days (T5), and 28 days (T6) thereafter. Relevant baseline data (demographic data, primary site of infection), clinical data (disease severity scores, such as Simplified Acute Physiology Score (SAPS II), Sequential Organ Failure Assessment Score (SOFA) and Acute Physiology Health Evaluation score (APACHE II), surgical procedures, antifungal therapy, outcome parameters) as well as routine infection parameters (e.g. leukocytes, C-reactive protein (CRP), procalcitonin (PCT), body temperature) were collected.

### 4.2. Immunoassays

Plasma concentrations of BG were measured using the Glucatell^®^-Kit (Pyroquant Diagnostik GmbH) according to the manufacturer’s instructions. Plasma concentrations of MR-proADM were measured using a TRACE (Time Resolved Amplified Cryptate Emission) technology in combination with a new sandwich immunoassay (Kryptor Compact Plus Analyser, BRAHMS, Hennigsdorf, Germany). In all patients, concentrations of GM were measured using an enzyme-linked immunoassay (Platelia^TM^ Aspergillus AG, Biorad, Munich) in plasma samples at all time points. Concentrations of GM in bronchoalveolar lavage fluid (BALF) were measured using the same technique, however only in selected cases of suspected invasive aspergillosis (IA). The following GM concentrations were used as cut-off values: Plasma > 0.5, BALF > 1.0.

### 4.3. Flowcytometry

Plasma concentrations of IL-2, -4, -6, -10, -17A, TNF-α, and IFN-γ were measured on a FACSVerse flow cytometer (BD Biosciences, Heidelberg, Germany) using a multiplex assay (Human Th1/Th2/Th17 Cytokine Kit, BD Biosciences, Heidelberg, Germany) according to the manufacturer’s instructions.

### 4.4. Clinical Microbiology

#### 4.4.1. Blood Culture

Blood culture testing at Heidelberg University Hospital was routinely performed as described elsewhere [109]. Whole blood samples were obtained via direct venipuncture (e.g., antecubital vein) applying sterile techniques, and 10 mL blood was inoculated to both an aerobic and an anaerobic liquid culture medium. Cultures were incubated for 5 days and positive cultures analyzed according to approved in-house hospital standard techniques, including identification by VITEK2 (Biomerieux, Nuertingen, Germany) or MALDI TOF (Bruker, Madison, WI, USA) and automated antimicrobial susceptibility testing (VITEK 2).

#### 4.4.2. Culture-Based Diagnostic Procedures in Tracheal Secretion, Wound Swabs, and Drainage Fluids

Briefly, tracheal aspirates and drainage fluids were streaked manually on Columbia (Becton, Dickinson, Franklin Lakes, NJ, USA), chocolate (bioMérieux SA, Marcy l’Etoile, France), MacConkey (bioMérieux SA, Marcy l’Etoile, France), Schaedler and kanamycin-vancomycin (Bi-plate, Becton, Dickinson, Franklin Lakes, NJ, USA) and chromogenic Candida agar (Becton, Dickinson, Franklin Lakes, NJ, USA), while wound swabs were inoculated semi-automated by PREVI Isola™ instrument on the same agar types. All plates were incubated at 37 °C in 5% CO_2_ for 24 to 48 h, except the Schaedler-KV bi-plates, which were incubated at 37 °C in an anaerobic chamber (GasPak; Becton, Dickinson, Franklin Lakes, NJ, USA) for 48 h as described [110]. Bacterial and fungal colonies were identified by MALDI TOF mass spectrometry and automated antibiotic susceptibility testing (AST) was performed on VITEK II instruments (bioMérieux SA, Marcy l’Etoile, France).

#### 4.4.3. Group Definitions

*Candida* spp. in the respiratory tract or in fluids from drainages were classified as colonization. Positive results in blood cultures, intraoperative swabs, and *Aspergillus* spp. in deep respiratory tract specimens with accompanying pulmonary infiltrates were classified as infection.

#### 4.4.4. Anti-*Candida*-Antibody Titer

*Candida albicans* specific IgM, IgA and IgG antibodies in serum were detected and quantified using Serion Enzyme-linked Immunosorbent Assay (ELISA) classic ™Candida albicans IgA/IgG/IgM (ESR 117A/G/M, Virion Serion, Wuerzburg, Germany) as described in the manufacturer’s instructions using a Behring ELISA Processor (BEP III, Siemens Healthcare Diagnostics, Marburg, Germany) [111].

### 4.5. Next Generation Sequencing (NGS)-Based Diagnostic Procedures

#### 4.5.1. Plasma Preparation and Nucleic Acid Isolation

Plasma was prepared from blood samples by centrifugation for 10 min at 292× *g* and 4 °C, snap frozen and stored at −80 °C until further processing. Nucleic acids were isolated from thawed plasma after a centrifugation step of 5 min at 16,000× *g* at 4 °C with the QIAsymphony sample preparation (SP) instrument (Qiagen, Hilden, Germany) and the QIAsymphony DSP Circulating DNA Kit according to the manufacturer’s protocol. To ensure preparation from equal volumes of 1000 µL, 1200 µL starting volume plasma was loaded per sample. There were the following variations from the protocol: if plasma volumes were below 1000 µL after centrifugation, the respective samples were excluded from the analysis. Plasma volumes of above 1000 µL were adjusted to 1200 µL with sterile phosphate buffered saline (Carl Roth, Karlsruhe, Germany). Nucleic acids were eluted in 60 µL molecular biology grade water (5 Prime, Hilden, Germany). Contamination controls were prepared following the same procedure, starting from 1200 µL molecular biology grade water and 1200 µL of sterile phosphate buffered saline, which were prepared accordingly. The cfDNA was quantified with the Qubit dsDNA HS Assay Kit (Life Technologies, Carlsbad, CA, USA) and quality was assessed with the HS NGS Fragment Analysis Kit and the Fragment Analyzer instrument (AATI).

#### 4.5.2. Preparation of NGS Libraries and Sequencing

Library preparation and sequencing were carried out as previously described [27] from 1 ng cfDNA using the Nextera XT library preparation kit (Illumina, San Diego, CA, USA), with a Biomek FXP liquid handling robot (Beckman Coulter, Brea, CA, USA). Sequencing of the libraries was performed on a HiSeq2500 (Illumina, San Diego, CA, USA), resulting in 33 million 100-bp single end reads, on average, per sample. The utilized raw data for NGS-based diagnostics is deposited in the European Nucleotide Archive under the following accession number: PRJEB21872.

#### 4.5.3. Bioinformatics

Bioinformatic processing, classification and SIQ score calculation was carried out as described in [27] (Table S4). Species hits were excluded according to the following criteria: (i) taxonomy in RefSeq database does show a clear species assignment; (ii) hit is a phage; (iii) bacterial/viral hits with less than ten or fungal hits with less than two normalized counts, respectively; (iv) p-value above 0.05; (v) hits belong to known contaminant genus according to Salter and colleagues [112] except SIQ score is above 500 and species previously described as a pathogen (labeled with contaminant tag a); (vi) species counts in more than 50% of all samples detected according to the complete investigated cohort, except SIQ score is above 500 and species previously described as a pathogen (labeled with contaminant tag b); (vii) all hits from *Stenotrophomonas maltophilia*, *Rhodococcus erythropolis*, *Ralstonia pickettii*, and *Herbaspirillum seropedicae* as they show a SIQ score of 500 or above in more than 25% of the samples according to the complete investigated cohort.

### 4.6. Gene Expression Analysis of Infected Reconstituted Human Epithelia (RHE)

#### 4.6.1. Cell Culture Model

The reconstituted human epithelia (RHE) was established with the vulvovaginal cell line A-431 in ThinCert cell culture inserts in a 6-well format with a pore size of 0.4 µm over eight days (Greiner, Germany). In 2 mL KBM medium supplemented with the Gold Bullet Kit inclusive of the provided antibiotics (Lonza, Basel, Switzerland), 5 × 10^6^ cells were disseminated per insert at day 0. An additional 2 mL of the same medium was added directly into the well. The cells were cultivated at 37 °C, 5% CO_2_ and 95% humidity. The medium was refreshed daily, and medium in the insert omitted from the fifth day onwards. On day seven, the antibiotics in the medium were exchanged with 0.05 mM CaCl_2_. On day eight and 4 h prior to the infection we provided the multilayered RHE with fresh medium in the well. For infection, we applied 1.5 × 10^7^ exponentially growing *Candida* cells in 2 mL supplemented KBM without antibiotics. Here, *Candida albicans* strain SC5314 and *Candida dubliniensis* strain CD36 were used. Experiments were performed in biological duplicates.

#### 4.6.2. RNA-Seq

RNA was isolated before infection and 60 and 180 min after infection from RHE infected with *C. albicans* and *C. dubliniensis*, and from uninfected RHE. For this, the medium in the insert was discarded and the RHE was washed with 1 mL phosphate buffered saline (pH 7.4). The cells were then lysed with 800 µL lysis buffer RLTplus (QIAGEN, Hilden, Germany), and supplemented with 1% *v*/*v* of β-ME by pipetting up and down ten times. A centrifugation step for 2 min at 16,000× *g* was included in the protocol to remove the *Candida* cells. Subsequent extraction of total RNA was then performed according to QIAGEN’s Purification of Total RNA from Animal and Human Tissues Protocol without additional cell disruption step, using the RNeasy Plus Mini Kit. RNA quality and quantity was evaluated using an Agilent 2100 Bioanalyzer with RNA 6000 Nano Chips (Santa Clara, CA, USA). cDNA libraries were prepared with 500 ng of total RNA and Illumina’s TruSeq RNA Sample Preparation v2 protocol. Sequencing was performed using an Illumina HiSeq2000. To assess the concentration and ensure appropriate size distribution (between 200–570 bp), cDNA libraries were checked using Bioanalyzer DNA 1000 chips. Sequencing run was carried out with paired-end 65 bp long reads following the manufacturer’s instructions with an average sequencing depth of 60 million paired-reads. Reads were mapped against the human reference genome assembly GRCh38.80 using NextGenMap (v. 0.4.12, http://cibiv.github.io/NextGenMap/) with default settings [113]. Downstream quantification of genes in raw read counts as well as in RPKM (=reads per kilobase of exon model per million mapped reads according to Mortazavi et al. [114]) was carried out exclusively with uniquely mapped read pairs using Gencode annotation v22 (Ensembl release 80) with the python script “rpkmforgenes.py” (http://sandberg.cmb.ki.se/rnaseq/). Identification of differentially expressed genes (DEGs) was done using Bioconductor package edgeR (version 3.4.2, https://bioconductor.org/packages/release/bioc/html/edgeR.html) with two biological replicates per condition based on raw read counts [115]. In order to reduce background signals, we applied stringent criteria considering only genes with an adjusted *p*-value (FDR, false discovery rate) < 0.001 and a log_2_ fold change ≤−1.5 or ≥1.5 as being significantly differentially regulated between two conditions (Table S2).The utilized raw data for RNA-Seq is deposited in European Nucleotide Archive under the following accession number: PRJEB21872.

### 4.7. Statistical Analyses

The resulting data were entered into an electronic database (Excel 2010; Microsoft Corp, Redmond, WA, USA) and evaluated using the SPSS software (Version 21.0; SPSS, Inc., Chicago, IL, USA). Categorical data were summarized using absolute and relative frequencies. Quantitative data were summarized using median with quartiles. The Kolmogorov-Smirnov test was applied to check for normal distribution. Due to non-normally distributed data, non-parametric methods for evaluation were used (Chi-square test for categorical data, Mann-Whitney *U* test for continuous data). Appropriate cut-off values for plasma levels of IL-17A as well as MR-proADM for the detection of a fungal infection were calculated using ROC analyses. A *p*-value < 0.05 was considered statistically significant. Concerning symbolism and higher orders of significance: * *p* < 0.05, ** *p* < 0.01, *** *p* < 0.001.

## 5. Conclusions

Fungal pathogens are very common in critically ill patients and are associated with an increased morbidity, necessitating a comprehensive, reliable and fast diagnosis. In this respect, an NGS-based diagnostic approach appeared to be suitable for the detection of fungal pathogens in plasma samples of patients with septic shock. Moreover, the fungal SIQ-score allowed for the identification of invasive mycoses even in those patients who have so far been classified as colonized (based on culture-based diagnostic procedures) and usually have not received prior antifungal therapy. The identification of patients with invasive mycoses can be further facilitated by plasmatic measurements of IL-17A as well as MR-proADM, which was furthermore identified to be an early target gene in response to epithelial infections with *Candida* spp. Accordingly, the implementation of MR-proADM and IL-17A measurements in routine diagnostics for the detection of invasive mycoses should be taken into account. Conversely, plasma levels of BG did not significantly differ between patients without any fungal pathogens, colonized as well as infected patents. Therefore, measurements of BG failed to be of value for the identification of invasive mycoses, although BG represents a member of the cell wall of most fungi and has previously been proposed as a biomarker for invasive fungal infections with *Candida* spp., *Aspergillus* spp. or *Pneumocystis jirovecii*.

## Figures and Tables

**Table 1:** Patient’s characteristics (*n* = 50).

Parameter	Unit	All Patients (*n* = 50)	Without Fungal Isolates (*n* = 17)	With Fungal Isolates (*n* = 33)	*p* for Patients without Fungal Isolates vs. Patients with Fungal Isolates
Gender male		38 (76)	11 (64.7)	27 (81.8)	0.160
Age	(years)	66 (61–75)	71 (64–80)	66 (59–74)	0.117
BMI	(kg/m^2^)	27.2 (24.4–30.9)	27.2 (25.7–34.9)	26.9 (23.1–30.9)	0.401
Postoperative peritonitis		31	9 (52.9)	22 (66.7)	0.206
initial operation					
Kidney		2 (4)	0 (0)	2 (6.1)	0.431
Liver		11 (22)	1 (2.1)	10 (30.3)	0.047 *
Pancreas		2 (10)	1 (5.9)	1 (3.0)	0.569
GIT		38 (76)	14 (82.4)	24 (72.7)	0.350
VAS		3 (6)	2 (11.8)	1 (3.0)	0.264
Others		12 (24)	3 (17.6)	9 (27.3)	0.350
≥48 h after hospital admission		25 (50)	7 (41.2)	18 (54.5)	0.276
NYHA 0-I		41 (82)	13 (76.4)	28 (84.8)	0.358
Diabetes mellitus		17 (34)	5 (29.4)	12 (36.3)	0.434
Arterial hypertension		34 (68)	12 (70.6)	22 (66.7)	0.520
Coronary heart disease		8 (16)	5 (29.4)	3 (9.1)	0.076
Chronic obstructive lung disease		10 (20)	5 (29.4)	5 (15.2)	0.204
Renal insufficiency		7 (14)	1 (5.9)	6 (18.2)	0.231
Renal replacement therapy		15 (30)	2 (11.8)	13 (39.4)	0.041 *
Liver cirrhosis		13 (26)	3 (17.6)	10 (30.3)	0.270
Oncological disease		33 (66)	11 (64.7)	22 (66.7)	0.566
APACHE II ^#^		30 (28–35)	32 (28–36)	30 (28–34)	0.491
SOFA ^#^		11 (10–14)	11 (10–14)	11 (10–14)	0.959
SAPS II ^#^		65 (49–75)	72 (48–75)	65 (51–72)	0.467
*Candida* colonization		22 (44)	0 (0)	22 (66.7)	---
*Candida* infection		10 (20)	0 (0)	10 (30.3)	---
Candidemia		3 (6)	0 (0)	3 (9.1)	---
*Aspergillus* spp.		1 (3)	0 (0)	1 (3.0)	---
Candida-Score		4 (4–4)	4 (4–4)	4 (4–4)	0.080
Ventilation duration	(h)	145.5 (67.3–450)	89 (46–145)	181 (77–682)	0.015 *
ICU length of stay	(days)	19.5 (12–44)	12 (3–17)	24 (15–46)	0.002 **
Hospital length of stay	(days)	44 (23.3–68.5)	24 (12–40)	51 (39–78)	0.007 **
Tracheotomy		14 (28)	2 (11.8)	12 (36.3)	0.063
Anastomosis leakage		24 (48)	7 (41.2)	17 (51.5)	0.347
Fascia dehiscence		12 (24)	2 (11.8)	10 (30.3)	0.134
90 day mortality		17 (34)	8 (47.1)	9 (27.3)	0.175
28 day mortality		11 (22)	7 (41.2)	4 (12.1)	0.025 *

Data are presented either as number (with the corresponding percentage value) or as median (with accompanying quartiles (Q1–Q3). Legend: BMI = Body mass index, GIT = gastro intestinal tract, VAS = vascular artery surgery, NYHA = New York Heart Association score, APACHE II = Acute Physiology Health Evaluation score, SAPS II = Simplified Acute Physiology Score, SOFA = Sequential Organ Failure Assessment score; ^#^ calculated at sepsis onset. Concerning symbolism and higher orders of significance: * *p* < 0.05, ** *p* < 0.01, --- : not calculated.

**Table 2:** Characteristics of patients with a fungal colonization or a fungal infection.

Parameter	Unit	Fungal Colonization (*n* = 22)	Fungal Infection (*n* = 11)	*p* for Patients with Fungal Colonization vs. Patients with Fungal Infection
Male		17 (77.3)	10 (90.1)	0.329
Age	(years)	66 (61–74)	65 (58–74)	0.355
BMI	(kg/m^2^)	25.3 (21.6–30.8)	27.4 (26–30.5)	0.925
Postoperative peritonitis		14 (63.6)	8 (72.7)	0.454
Initial operation				
Kidney		1 (4.5)	1 (9.1)	0.563
Liver		3 (13.6)	7 (63.6)	0.006 **
Pancreas		1 (4.5)	0 (0)	0.667
GIT		16 (72.7)	8 (72.7)	0.653
VAS		0 (0)	1 (9.1)	0.333
Others		5 (22.7)	4 (36.4)	0.333
≥48 h after hospital admission		15 (68.2)	3 (27.3)	0.031 *
NYHA 0-I		17 (77.3)	11 (100)	0.111
Diabetes mellitus		8 (36.4)	4 (36.4)	0.653
Arterial hypertension		15 (68.2)	7 (63.6)	0.546
Coronary heart disease		2 (9.1)	1 (9.1)	0.748
Chronic obstructive lung disease		5 (22.7)	0 (0)	0.111
Renal insufficiency		5 (22.7)	1 (9.1)	0.329
Renal replacement therapy		8 (36.4)	5 (45.5)	0.446
Liver cirrhosis		3 (13.6)	7 (63.6)	0.006 **
Oncological disease		14 (63.6)	8 (72.7)	0.454
APACHE II ^#^		30 (29–34)	29 (28–33)	0.396
SOFA ^#^		11 (10–13)	14 (11–15)	0.044 *
SAPS II ^#^		61 (44–72)	68 (57–77)	0.336
Candida-Score		4 (4–4)	4 (4–4)	1.0
Ventilation duration	(h)	148.5 (74–239.3)	600 (424.5–944)	0.040 *
Tracheotomy		2 (9.1)	8 (72.7)	0.002 **
Fascia dehiscence		3 (13.6)	7 (63.6)	0.006 **
Anastomosis leakage		11 (50)	6 (54.5)	0.549
ICU length of stay	(days)	21 (13.5–43.5)	38 (25.5–64)	0.082
Hospital length of stay	(days)	50 (34.5–68.5)	53 (47.5–88)	0.418
90 day mortality		4 (18.2)	5 (45.5)	0.120
28 day mortality		3 (13.6)	1 (9.1)	0.593

Data are presented either as number (with the corresponding percentage value) or as median (with accompanying quartiles (Q1–Q3). Legend: BMI = Body mass index, GIT = gastro intestinal tract, VAS = vascular artery surgery, NYHA = New York Heart Association score, APACHE II = Acute Physiology Health Evaluation score, SAPS II = Simplified Acute Physiology Score, SOFA = Sequential Organ Failure Assessment score, ^#^ calculated at sepsis onset. Concerning symbolism and higher orders of significance: * *p* < 0.05, ** *p* < 0.01.

**Table 3:** Characteristics of patients with or without a fungal infection.

Parameter	Unit	Without Fungal Infection (*n* = 39)	Fungal Infection (*n* = 11)	*p* for Patients without Fungal Infection vs. Patients with Fungal Infection
Gender male		28 (71.8)	10 (90.1)	0.184
Age	(years)	66 (63–76)	65 (58–74)	0.460
BMI	(kg/m^2^)	27.2 (23.2–33.2)	27.4 (26–30.5)	0.582
Postoperative peritonitis		23 (58.9)	8 (72.7)	0.322
initial operation				
Kidney		1 (2.6)	1 (9.1)	0.395
Liver		4 (10.3)	7 (63.6)	0.001 ***
Pancreas		2 (5.1)	0 (0)	0.605
GIT		30 (76.9)	8 (72.7)	0.528
VAS		2 (5.1)	1 (9.1)	0.534
Others		8 (20.5)	4 (36.4)	0.240
≥48 h after hospital admission		22 (56.4)	3 (27.3)	0.085
NYHA 0-I		30 (76.9)	11 (100)	0.115
Diabetes mellitus		13 (33.3)	4 (36.4)	0.560
Arterial hypertension		27 (69.2)	7 (63.6)	0.495
Coronary heart disease		7 (17.9)	1 (9.1)	0.430
Chronic obstructive lung disease		10 (25.6)	0 (0)	0.062
Renal insufficiency		6 (15.4)	1 (9.1)	0.604
Renal replacement therapy		10 (25.6)	5 (45.5)	0.184
Liver cirrhosis		6 (15.4)	7 (63.6)	0.003 **
Oncological disease		25 (64.1)	8 (72.7)	0.440
APACHE II ^#^		31 (28–36)	29 (28–33)	0.335
SOFA ^#^		11 (10–13)	14 (11–15)	0.081
SAPS II ^#^		62 (47–74)	68 (57–77)	0.519
Candida-Score		4 (4–4)	4 (4–4)	0.881
*Candida* colonization		22 (56.4)	0 (0%)	---
*Candida* infection		0 (0%)	10 (90.1)	---
Candidemia		0 (0%)	3 (27.3)	---
*Aspergillus* spp.		0 (0%)	1 (9.1)	---
Ventilation duration	(h)	120 (60–200)	600 (424.5–944)	0.007 **
ICU length of stay	(days)	16 (10–28.5)	38 (25.5–64)	0.008 **
Hospital length of stay	(days)	40 (21.5–63)	53 (47.5–88)	0.075
Tracheotomy		4 (10.3)	8 (72.7)	0.001 ***
Anastomosis leakage		18 (46.2)	6 (54.5)	0.440
Fascia dehiscence		13 (33.3)	7 (63.6)	0.002 **
90 day mortality		12 (30.8)	5 (45.5)	0.293
28 day mortality		10 (25.6)	1 (9.1)	0.232

Data are presented either as number (with the corresponding percentage value) or as median (with accompanying quartiles (Q1–Q3). Legend: BMI = Body mass index, GIT = gastro intestinal tract, VAS = vascular artery surgery, NYHA = New York Heart Association score, APACHE II = Acute Physiology Health Evaluation score, SAPS II = Simplified Acute Physiology Score, SOFA = Sequential Organ Failure Assessment score; ^#^ calculated at sepsis onset. Concerning symbolism and higher orders of significance: ** *p* < 0.01, *** *p* < 0.001, --- : not calculated.

**Table 4:** First- and second-line antifungal therapy (*n* = 21).

First-Line Antifungal Therapy	*n* = 21 (%)
Fluconazole	7 (33.3)
Caspofungin	13 (61.9)
Liposomal amphotericin B	1 (4.8)
Used as empiric therapy	7 (33.3)
Fluconazole	1 (14.3)
Caspofungin	6 (85.7)
Change in antifungal therapy	5 (23.9)
**Second-Line Antifungal Therapy**	***n* = 5 (%)**
Caspofungin	4 (80.0)
Fluconazole	1 (20.0)

Data are given as number (with the corresponding percentage value).

## Supplementary Materials

Supplementary materials can be found at www.mdpi.com/1422-0067/18/8/1796/s1.

## Abbreviations

AATI	HS NGS Fragment Analysis Kit and the Fragment Analyzer instrument
ADM	Adrenomedullin
APACHE II	Acute Physiology Health Evaluation score
ARDS	Acute respiratory distress syndrome
AST	automated antibiotic susceptibility testing
BALF	Bronchoalveolar lavage fluid
BC	Blood culture
BMI	Body mass index
BG	β-d-glucan
bp	Base pair
*C. albicans*	*Candida albicans*
*C. glabrata*	*Candida glabrata*
*Candida* spp.	*Candida* species
cfDNA	Cell-free DNA
CFG	Caspofungin
CLR	C-type leptin receptors
COPD	Chronic obstructive pulmonary disease
CRP	C-reactive protein
CVC	Central venous catheter
DC	Dendritic cells
DNA	Deoxyribonucleic acid
DOR	Doripenem
ELISA	Enzyme-linked Immunosorbent Assay
EORTC	European Organisation for Research and Treatment of Cancer
FLC	Fluconazole
GIT	Gastro intestinal tract
GM	Galactomannan
IA	Invasive Aspergillosis
ICU	Intensive Care Unit
IPM:CIL	Imipenem/Cilastatin
INF-γ	Interferon gamma
IL	Interleukin
IL-17A	Interleukin-17A
MEM	Meropenem
MR-proADM	Mid-regional proadrenomedullin
MSG	National Institute of Allergy and Infectious Disease Mycoses Study Group
na	Not available
nd	Not detectable
NGS	Next-generation sequencing
NYHA	New York Heart association score
PAMP	aminotemrinal peptide of proadrenomedullin
PAMPs	Pathogen associated molecular patterns
PCR	Polymerase chain reaction
PCT	Procalcitonin
PRR	Pattern recognition receptors
RHE	Reconstituted human epithelia
RPKM	Reads per kilobase of exon model per million
SAPS II	Simplified Acute Physiology Score
SIQ	Sepsis indicating quantifier
SOFA	Sequential Organ Failure Assessment Score
spp.	Species
T_reg_	Regulatory T-cell
TBC	Inhaled Tobramycine
TLR	Toll-like receptor
TRACE	Time Resolved Amplified Cryptate Emission
TS	Tracheal secretion
TZP	Piperacilline/Tazobactam
VAC	Vancomycin
VAS	Vascular artery surgery
