# Supplementary material for: Immune-Response Patterns and Next Generation Sequencing Diagnostics for the Detection of Mycoses in Patients with Septic Shock—Results of a Combined Clinical and Experimental Investigation
Source: Int J Mol Sci. 2017 Aug 18;18(8):1796. doi: 10.3390/ijms18081796 (PMC5578184; doi:10.3390/ijms18081796)
Supplement: Supplementary file 1 [file ijms-18-01796-s001.zip › Supplemental Table S3.docx]

**Supplemental Table S3.** Plasma levels of different infection and inflammation markers.

|  | T0 | | | T1 | | | T2 | | | T3 | | | T4 | | | T5 | | | T6 | | |
| --- | --- | --- | --- | --- | --- | --- | --- | --- | --- | --- | --- | --- | --- | --- | --- | --- | --- | --- | --- | --- | --- |
|  | without fungal isolates | fungal colonization | fungal infection | without fungal isolates | fungal colonization | fungal infection | without fungal isolates | fungal colonization | fungal infection | without fungal isolates | fungal colonization | fungal infection | without fungal isolates | fungal colonization | fungal infection | without fungal isolates | fungal colonization | fungal infection | without fungal isolates | fungal colonization | fungal infection |
| leukocytes 1/nl | 8.37  (7.12-  14.47) | 13.43  (3.17-  25.75) | 13.12  (10.78-  19.52) | 19.76  (7.93-  24.33) | 19.76  (13.14-  28.69) | 17.27  (14.81-  25.31) | 16.87  (8.03-  22.03) | 21.42  (17.23-  28.07) | 15.08  (11.16-  26.12) | 14.66  (11.07-  17.03) | 15.98  (13.23-  23.16) | 16.87  (11.31-  24.82) | 9.35  (7.88-  10.79) | 11.55  (9.50-  17.93) | 15.62  (10.80-  19.31) | 8.01  (6.58-  10.83) | 11.71  (9.90-  19.15) | 23.09  (11.43-  29.46) | 8.84  (7.97-  9.70) | 12.97  (10.52-  15.44) | 20.49  (14.87-  22.26) |
| + | 0.532 | | | 0.082 | | | **0.000***** | | | 0.177 | | | 0.421 | | | **0.000***** | | | **0.000***** | | |
| ++ | 0.175 | | | 0.687 | | | 0.910 | | | .0314 | | | **0.050*** | | | 0.117 | | | 0.333 | | |
| +++ | 0.778 | | | 0.815 | | | 0.168 | | | 0.917 | | | 0.452 | | | 0.227 | | | 0.456 | | |
| CRP (mg/l) | 190.9  (123.7-  268.5) | 201.3  (151.7-  293.4) | 167.1  (157.5-  236.0) | 261.0  (204.5-  307.2) | 275.7  (218.0-  335.3) | 247.5  (184.7-  282.3) | 294.5  (214.0-  337.5) | 252.8  (170.3-  315.8) | 224.0  (172.9-  303.1) | 141.0  (98.8-  189.4) | 138.9  (116.0-  176.1) | 132.6  (118.1-  156.1) | 125.1  (84.2-  170.8) | 146.7  (73.0-  156.3) | 130.6  (77.8-  168.6) | 65.6  (58.3-  88.0) | 113.0  (91.2-  144.8) | 134.0  (111.1-  151.5) | 69.7  (69.7-  69.8) | 64.3  (39.2-  97.1) | 102.8  (64.2-  147.9) |
| + | 0.180 | | | **0.000***** | | | **0.002**** | | | 0.096 | | | 0.301 | | | **0.003**** | | | **0.040*** | | |
| ++ | 0.942 | | | 0.647 | | | 0.392 | | | 0.809 | | | 1.000 | | | 0.492 | | | 0.524 | | |
| +++ | 0.693 | | | 0.531 | | | 0.938 | | | 1.000 | | | 0.825 | | | 0.563 | | | 0.165 | | |
| PCT  (ng/ml) | 3.06  (1.21-  34.30) | 7.71  (4.04-  14.03) | 17.60  (6.88-  31.97) | 6.29  (2.88-  28.26) | 4.09  (1.86-  23.97) | 19.09  (7.91-  38.65) | 3.49  (1.88-  36.69) | 3.88  (3.32-  4.60) | 21.29  (16.04-  26.53) | 0.32  (0.15-  0.60) | 0.57  (0.36-  1.00) | 5.38  (4.30-  5.55) | 0.00  (0.00-  0.00) | 1.21  (0.60-  1.81) | 1.92  (1.70-  2.15) | 0.00  (0.00-  0.00) | 0.00  (0.00-  0.00) | 0.82  (0.62-  1.02) | 0.00  (0.00-  0.00) | 0.00  (0.00-  0.00) | 2.53  (1.92-  3.14) |
| + | 0.754 | | | 0.055 | | | 0.200 | | | 0.222 | | | 1.000 | | | 0.111 | | | 0.133 | | |
| ++ | 0.492 | | | 0.524 | | | 1.000 | | | **0.029*** | | | 1.000 | | | 1.000 | | | 1.000 | | |
| +++ | 0.297 | | | 0.254 | | | 0.200 | | | **0.008**** | | | 1.000 | | | 1.000 | | | 1.000 | | |
| TNF-α (pg/ml) | 0.04  (0.00-1.42) | 0.00  (0.00-0.89) | 0.76  (0.00-1.47) | 0.00  (0.00-0.56) | 0.16  (0.00-1.68) | 0.04  (0.00-1.85) | 0.04 (0.00-1.51) | 0.52  (0.00-1.85) | 1.06 (0.09-1.95) | 0.00 (0.00-0.00) | 0.00  (0.00-0.00) | 0.18 (0.00-0.33) | 0.00 (0.00-0.00) | 0.00  (0.00-0.00) | 0.00 (0.00-0.39) | 0.00 (0.00-0.00) | 0.00  (0.00-0.00) | 0.58 (0.00-1.57) | 0.00 (0.00-0.00) | 0.00  (0.00-0.00) | 1.19 (0.00-3.64) |
| + | 0.726 | | | 0.2010 | | | 0.596 | | | 0.897 | | | 0.637 | | | 0.900 | | | 1.00 | | |
| ++ | 0.487 | | | 0.403 | | | 0.277 | | | **0.013*** | | | 0.113 | | | 0.183 | | | 0.333 | | |
| +++ | 0.357 | | | 0.755 | | | 0.553 | | | **0.008**** | | | 0.136 | | | 0.067 | | | 0.094 | | |
| IL-2  (pg/ml) | 0.06  (0.00-0.68) | 0.00  (0.00-0.88) | 0.00  (0.00-2.69) | 0.16 (0.00-0.45) | 0.20  (0.00-2.05) | 0.47  (0.00-1.84) | 0.06 (0.00-0.13) | 0.00  (0.00-1.55) | 0.45 (0.14-1.69) | 0.04 (0.00-0.08) | 0.00  (0.00-0.04) | 0.37 (0.00-0.75) | 0.00 (0.00-0.13) | 0.00  (0.00-0.00) | 0.18 (0.10-0.88) | 0.00 (0.00-0.01) | 0.00  (0.00-0.04) | 0.88 (0.26-1.64) | 0.06 (0.04-0.08) | 0.14  (0.06-0.20) | 1.54 (0.12-2.43) |
| + | 0.878 | | | 0.516 | | | 0.705 | | | 0.187 | | | 0.357 | | | 0.704 | | | 0.533 | | |
| ++ | 0.789 | | | 0.789 | | | 0.134 | | | 0.169 | | | 0.063 | | | 0.067 | | | 0.333 | | |
| +++ | 0.638 | | | 0.907 | | | 0.525 | | | **0.046*** | | | **0.010**** | | | **0.003**** | | | 0.121 | | |
| IL-4  (pg/ml) | 0.00  (0.00-0.24) | 0.08  (0.00-0.96) | 0.00  (0.00-1.30) | 0.00 (0.00-0.21) | 0.44  (0.00-2.53) | 0.44  (0.06-0.91) | 0.00 (0.00-0.54) | 0.00  (0.00-1.62) | 0.37 (0.00-0.82) | 0.00 (0.00-0.00) | 0.00  (0.00-0.00) | 0.18 (0.07-0.62) | 0.00 (0.00-0.00) | 0.00  (0.00-0.01) | 0.13  (0.0-0.90) | 0.00 (0.00-0.00) | 0.00  (0.00-0.00) | 0.31 (0.05-1.05) | 0.00 (0.00-0.00) | 0.01  (0.00-0.05) | 0.79 (0.22-1.55) |
| + | 0.362 | | | 0.118 | | | 0.762 | | | 0.754 | | | 0.522 | | | 0.900 | | | 0.400 | | |
| ++ | 0.517 | | | 0.066 | | | 0.649 | | | **0.001***** | | | **0.024*** | | | 0.117 | | | 0.111 | | |
| +++ | 0.985 | | | 0.969 | | | 0.899 | | | **0.001***** | | | **0.043*** | | | **0.014*** | | | **0.040*** | | |
| IL-6  (pg/ml) | 12815.5 (1104.4-22524.8) | 1944.2 (596.4-5603.9) | 8414.9 (3352.8-13318.1) | 460.9 (140.8-1005.00) | 209.0 (104.3-468.2) | 359.3 (202.7-1016.8) | 92.3 (57.9-246.0) | 57.3  (24.7-149.1) | 80.6 (41.9-157.9) | 20.0  (3.3-132.4) | 30.6  (21.1-97.1) | 142.9 (93.8-261.1) | 11.7  (3.0-28.9) | 18.3  (11.7-185.4) | 138.2 (74.7-268.6) | 4.7  (3.9-6.5) | 21.9  (12.0-46.1) | 61.2 (11.9-144.3) | 3.2 (2.9-3.5) | 13.9  (6.6-28.2) | 54.1 (41.7-161.4) |
| + | 0.279 | | | 0.278 | | | 0.323 | | | 0.494 | | | 0.169 | | | **0.014*** | | | 0.089 | | |
| ++ | 0.817 | | | 0.936 | | | 0.865 | | | 0.051 | | | **0.006**** | | | **0.033*** | | | 0.056 | | |
| +++ | **0.006**** | | | **0.048*** | | | 0.180 | | | **0.031*** | | | 0.152 | | | 0.643 | | | **0.004**** | | |
| IL-10 (pg/ml) | 14.63 (4.87-53.78) | 15.08  (6.19-24.90) | 18.74 (8.12-86.42) | 4.47 (1.45-5.87) | 5.31  (2.05-10.33) | 6.61  (4.22-11.03) | 2.39 (1.13-4.86) | 3.80  (0.93-6.73) | 4.10 (2.25-6.17) | 0.59 (0.18-1.17) | 0.57  (0.39-1.30) | 1.54 (0.63-2.89) | 0.16 (0.11-0.51) | 0.25  (0.11-0.60) | 1.19 (0.96-2.12) | 0.03 (0.01-0.06) | 0.35  (0.18-0.42) | 1.45 (1.26-2.88) | 0.02 (0.01-0.03) | 0.31  (0.13 -0.41) | 2.53 (1.27-3.02) |
| + | 0.922 | | | 0.516 | | | 0.762 | | | 0.449 | | | .0637 | | | 0.082 | | | 0.267 | | |
| ++ | 0.746 | | | 0.095 | | | 0.392 | | | **0.032*** | | | **0.001***** | | | 0.067 | | | 0.056 | | |
| +++ | 0.866 | | | 0.254 | | | 0.420 | | | 0.061 | | | **0.002**** | | | **0.030*** | | | **0.006**** | | |
| IFN-γ (pg/ml) | 0.00  (0.00-0.33) | 0.00  (0.0-0.76) | 0.00  (0.00-2.20) | 0.03  (0.00-0.20) | 0.14  (0.00- 1.82) | 1.37  (0.07-2.94) | 0.00  (0.00 -0.21) | 0.00  (0.00-  2.04) | 0.81 (0.00-4.31) | 0.00 (0.00-0.32) | 0.00  (0.00-0.08) | 1.09 (0.08-1.49) | 0.00 (0.00-0.00) | 0.00  (0.00-0.00) | 0.26 (0.17-2.51) | 0.00 (0.00-0.00) | 0.00  (0.00-0.08) | 1.86 (0.00-3.64) | 0.00 (0.00-0.00) | 0.00  (0.00-0.12) | 3.50 (0.17-4.45) |
| + | 0.747 | | | 0.309 | | | 0.791 | | | 0.754 | | | 0.718 | | | 0.364 | | | 0.533 | | |
| ++ | 0.430 | | | 0.051 | | | 0.134 | | | **0.027*** | | | **0.006*** | | | 0.183 | | | 0.111 | | |
| +++ | 0.638 | | | 0.434 | | | 0.328 | | | **0.006**** | | | **0.004*** | | | 0.157 | | | **0.029*** | | |

**Data are presented as median with accompanying quartiles (Q1, Q3).**

Legends: + = patients without fungal isolates vs. colonized patients, ++ = patients without fungal isolates vs. infected patients, +++ = colonized vs. infected patients. Plasma samples were collected at the onset of septic shock (T0), and 1 day (T1), 2 days (T2), 7 days (T3), 14 days (T4), 21 days (T5) and 28 days (T6) afterwards. p < 0.05: *, p < 0.01: **, p < 0.001: ***.
