# Supplementary material for: Immune-Response Patterns and Next Generation Sequencing Diagnostics for the Detection of Mycoses in Patients with Septic Shock—Results of a Combined Clinical and Experimental Investigation
Source: Int J Mol Sci. 2017 Aug 18;18(8):1796. doi: 10.3390/ijms18081796 (PMC5578184; doi:10.3390/ijms18081796)
Supplement: Supplementary file 1 [file ijms-18-01796-s001.zip › Supplemental Table S4.docx]

**Supplemental Table S4.** Candia-Score by Leon et al. (Crit Care Med 2006; 34: 730–737)

| Multifocal *Candida* spp. colonization (1 point) |  |
| --- | --- |
| Total parenteral nutrition (1 point) |  |
| Surgery on ICU admission (1 point) |  |
| Severe sepsis (2 points) |  |
| **Total** |  |
